# Divergent Evolutional Mode and Purifying Selection of the* KIT* Gene in European and Asian Domestic Pig Breeds

**DOI:** 10.1155/2018/8932945

**Published:** 2018-08-19

**Authors:** Lili Niu, KeYu Shi, Jing-Jing Xie, Sen Liu, Tao Zhong

**Affiliations:** Farm Animal Genetic Resources Exploration and Innovation Key Laboratory of Sichuan Province, College of Animal Science and Technology, Sichuan Agricultural University, Chengdu 611130, China

## Abstract

The recent geographic expansion of wild boars and the even more recent development of numerous domestic pigs have spurred exploration on pig domestic origins. The porcine* KIT* gene has been showed to affect pleiotropic effects, blood parameters, and coat colour phenotypes, especially the white colour phenotype formation in European commercial breeds. Here, we described the use of SNPs to identify different selection patterns on the porcine* KIT *gene and the phylogenetic relationships of the inferred haplotypes. The phylogenetic tree revealed four clades in European and Asian wild and domestic pigs: two major clades with European and Asian origins and one minor clade with Iberian origins as well as the other minor clade in Asia, consistent with the major introgression of domestic Asian pigs in Europe around 18th -19th century. The domestication history of pigs, which occurred in the domestication centers (Europe and Asia), has also been demonstrated by mtDNA analysis. Furthermore, both Asian and European domestic pigs evolved under purifying selection. This study indicated that domestic pigs in Europe and Asia have different lineage origins but the porcine* KIT* gene was undergoing a purifying selection during their evolutional histories.

## 1. Introduction

Coat colour variation in animals has been a considerable research hotspot in animal genetics and breeding. Documentation on coat colour can be traced back over 5,000 years ago to Mesopotamia [1]. Numerous mutations accumulated under the effects of natural and human selection, which contributed to the formulation of variations in the coat colour of animal species. Pigmentation in mammals is determined by the amount and distribution of two types of melanin, eumelanin (black/brown), and phaeomelanin (red/yellow), for which production and relative amounts are mainly controlled by the* agouti* and* extension* loci [2], which encode the agouti signalling protein (*ASIP*), and to the melanocortin receptor 1 (*MC1R*), respectively [3, 4]. However, the most studied and reported target gene is the* mast/stem cell growth factor receptor* (*KIT* gene) in porcine coat colour genetics. The* KIT* gene is located in the* Dominant White* locus and is confirmed to be responsible for several common colour variants in pigs, such as Dominant White, patch, spotting, belt, and roan [5–12].

The* KIT* gene encodes the* mast/stem cell growth factor receptor*, which is a cytokine receptor expressed on the surface of haematopoietic stem cells as well as other cell types [13]. This protein is a type 3 transmembrane receptor for the mast cell growth factor (*MGF*, also known as the stem cell factor). It contains an extracellular domain composed of five immunoglobulin domains, a single transmembrane domain, a juxtamembrane domain, and an intracellular protein kinase domain that is interrupted by an insertion of approximately 80 amino acids [14]. The protein has the potential to participate in multiple signalling pathways, which accounts for its important role in the control of cell differentiation, survival, and motility and acts as an essential survival factor for the migration and proliferation of melanoblasts [15]. Mutations in the* KIT* gene are associated with gastrointestinal stromal tumours, mast cell disease, and piebaldism in human [16]. In mice, loss-of-function mutations are associated with limited white spotting in heterozygotes, but the mutations are often lethal or sublethal in the homozygous condition due to their effects on haematopoiesis [17]. Variant coat colour patterns in horses also derive from the* KIT* gene mutations [18, 19].

Up to now, five alleles have been identified in the* KIT* gene, the recessive *i* allele (wild-type) present in wild boar and coloured pigs (Duroc, Asian black pigs), the* I*^*P*^ allele for the patch phenotype (Pietrain), the* I*^*Be*^ allele for the belt phenotype (Hampshire), the* I*^*Rn*^ allele for the roan/gray coat colour, and the major dominant *I* allele (*I*^*1*^,* I*^*2*^,* I*^*3*^, or* I*^*L*^) for the fully dominant white coat colour especially in European commercial breeds such as Landrace and Large White. The origin, domestication, and distribution of the alleles in the porcine* KIT* gene likely present an independent geographic trend. Subsequently, the European pigs harboured a normal copy number of the* KIT* gene (*i* and* I*^*Be*^ alleles) as well as duplicated copy numbers (*I*^*1*^,* I*^*2*^,* I*^*3*^, and* I*^*P*^ alleles), while only the normal copy number was identified in Asian pigs until now. Furthermore, none of the* Dominant White* alleles were identified in one Chinese white pig breed [20]. However, studies on the* KIT* gene were limited to only a few of pig breeds [6, 10, 11, 20, 21].

In this study, we aimed to investigate the DNA polymorphisms and haplotype distribution of the porcine* KIT* gene in 17 Asian wild and domestic breeds, as well as several European wild and domestic breeds. We also compared the divergent evolutional mode of the* KIT* gene and addressed which kinds of selection occurred in Asian and European domestic pigs during the process of domestication.

## 2. Materials and Methods

### 2.1. Animal Materials

A total of 44 unrelated pigs from 22 domestic breeds were analyzed in this study (15 Asian domestic breeds and 7 European domestic breeds, Supplementary Table 1). In addition, 12 European wild boars, two Chinese wild boars from different regions, and 8 Korean wild boars from 4 subgroups [22] were also included. Genomic DNA was extracted from blood by standard methods or from hair roots by Chelex extraction.

### 2.2. Detection of Polymorphisms in the Dominant White Locus

A total of 17 PCR primer sets were designed using Primer Premier 5 to amplify the 21 exon regions of the* KIT* gene. To identify exon boundaries, blast analysis was performed to compare the porcine* KIT* cDNA sequences (FJ938289) with the working graft sequence of* Sus scrofa* (AC141857.2). The information on primers is listed in Table 1. PCR was carried out in a 25-*μ*L reaction volume containing 40–50 ng of template DNA, 10 × PCR buffer, 100 *μ*M of dNTP, 10 pmol of each forward and reverse primers, and 1 unit of Taq DNA polymerase (GenetBio, Korea). The PCR program included an initial denaturation step of 95°C for 5 min followed by 40 cycles of 94°C for 30 s, 50-66°C for 30 s, and 72°C for 40 s, and a final extension step of 72°C for 5 min in a PTC-200 Programmable Thermal Controller (MJ Research, Inc., USA). PCR products were purified with GELaseTM Agarose Gel-Digesting Preparation kit (Epicenter, USA) and then were directly sequenced using the Big Dye terminator chemistry on the ABI 3130XL DNA Analyzers (PE Applied Biosystems, USA). Ambiguous positions were verified by resequencing. To determine if any contamination was present, blank PCR controls were performed throughout this study.

### 2.3. Sequencing of the Mitochondrial DNA D-Loop Region

According to the highly conserved tRNA-Pro and tRNA-Phe regions within the porcine mtDNA, one primer set was designed to amplify the D-Loop region as described in [22]. Purified PCR products were performed by direct sequencing using the forward PCR primer on the ABI3130XL DNA Analyzers.

### 2.4. Data Analysis

Nucleotide diversity (*π*), haplotype diversity (H_d_), and Watterson's theta estimator were calculated by the DNASP v. 4.0 [23]. To estimate the effect of selection, Tajima's D, Fu and Li's D^*∗*^, and F^*∗*^ were also estimated by the DNASP. Confidence intervals for Tajima's D and Fu and Li's D^*∗*^ and F^*∗*^ values were obtained by generating 1000 independent coalescent simulations assuming no recombination. The synonymous (silent) and nonsynonymous (nonsilent) nucleotide substitution rates of gene sequences were estimated by the modified Nei-Gojobori method descried by [24]. The MEGA v. 4.0 program was used for the evolutionary analysis [25].

Phylogenetic analyses of the* KIT* haplotypes were conducted by MrBayes 3.1 [26, 27] and MEGA, respectively [25]. A consensus tree was produced by MrBayes with default priors, such as the numbers of iterations and the sampling frequency, assumed after a pilot run with 1 million iterations. The main analysis was run until the average standard deviation of split frequencies fell below 0.01. Of the sampled parameter values, 25% were summarized to output a cladogram with the posterior probabilities for each split and a phylogram with mean branch lengths. The consensus tree was illustrated by FigTree3 (http://tree.bio.ed.ac.uk/software/figtree/). A neighbour-joining (NJ) tree was constructed with MEGA under Kimura's two-parameter model. Standard errors were obtained from 1000 bootstrap replicates.

The relationships of haplotypes were estimated by the parsimonious median-joining method and visualized using Network 4.5.1.6 and Network Publisher 1.2.0.0 (http://www.fluxus-engineering.com). Nucleotide weighting (*ω*) was adjusted to reflect the difference in mutational frequencies between transversions (*ω* = 20) and transitions (*ω* = 10).

## 3. Results and Discussion

### 3.1. Genetic Characteristics of the Porcine KIT Gene

The polymorphism sites in the whole coding region of the* KIT* gene were identified by direct sequencing among wild boars and six domestic breeds (Landrace, Large White, Hampshire, Berkshire, Duroc, and Korean native pigs). Subsequently, the variable sites were checked in the other individuals. A total of 28 polymorphisms including one indel event (intron18: g.29_32delAGTT) were obtained by a multiple alignment of the assembled 66 individual sequences using Clustal W [28]. Four novel mutations were revealed in this study, and one of them was missense mutation (c.583 G>A, Val195Met). A small proportion of the SNPs (5 out of 27) were missense mutations, while the others were sense mutations (Supplementary Table 1). A high degree of homozygosity was detected in domestic breeds compared to wild boars originating from either Europe or Asia. Asian domestic pigs also harboured higher diversities than European domestic pigs. It could be explained by human selection for breed-specific formation and later inbreeding for fixing the characteristics of a breed. The European domestic pigs had endured high human selections on coat colour, high performance, lean meat content, and so on. Furthermore, it is also indicated that Berkshire had been introgressed by Asian domestic pigs, because several SNPs (c.583 G>A, c.1863 C>T, c.1899 A>G, c.2199 C>T, c.2601 C>T, c.2661 C>T, and c.2748 C>T) were homozygous in European wild boar, while those were heterozygous in Berkshire, which contains the SNPs in Asian pigs (VT, BM, and XZ). This was consistent with previous studies, which documented introgression of Asian pigs that had occurred during the 18th and early 19th centuries [29–31]. The present study investigated the genetic variations of the* KIT* gene in wild and domestic pigs from Asia and Europe. It was noted that none of the 27 identified SNPs had given any effect of a camouflaged coat in either Asian or European wild boars. Even 5 of 27 were missense mutations, but Asian and European wild boars did not display any difference in phenotypic or physiological characters. Furthermore, no fixed mutation has been identified as the distinguishing SNP, which could separate the wild boar from domestic breeds.

### 3.2. The Neutrality Indices and Selection at the Porcine KIT Gene

**Figure 1:**
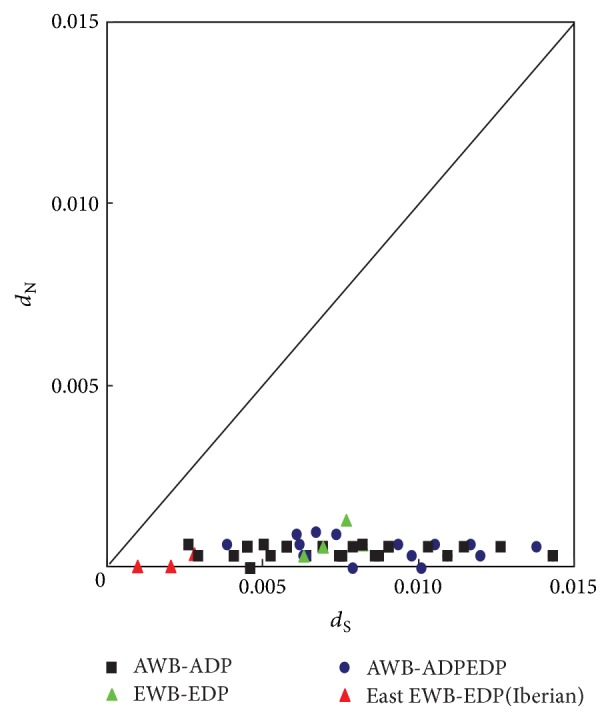
Pairwise synonymous (*dS*) and nonsynonymous (*dN*) substitutions of the* KIT* gene among wild and domestic pigs.

Comparison of variations at the* KIT* gene in the identified four subgroups is presented in Table 2. The values of neutrality indices of three branches (G 1, G 2, and G 4) were quite close to zero, except G3 (AWB-ADPEDP). The higher values that occurred in G3 might have resulted from crossbreeding, which occurred in BK, XZ, and VT. From the investigated European domestic pigs, clearly lower polymorphisms were revealed in comparison with European wild boars. Meanwhile, variation sites were mainly located in four exon regions of the* KIT* gene (exons 5, 6, 19, and 20). It could be explained by the unique breeding history of the European pigs, the majority of which were commercial breeds, except the Iberian pig, which was selected and developed strongly under the effects of human selection. Interestingly, Asian pigs displayed distinct mutations compared with those in European pigs. However, no more novel mutations were detected between Asian wild and domestic pigs, and the polymorphisms displayed a slight decrease in Asian domestic pigs. Aspects of selective pressures acting on the* KIT* gene were estimated by an analysis of the rates of synonymous, silent (*d*_S_), and nonsynonymous or nonsilent (*d*_N_) nucleotide substitutions among wild and domestic pigs from Asia and Europe (Figure 1). All the values of *d*_N_ revealed were well below the values of *d*_S_; furthermore, no obvious differences were detected among wild boars and domestic pigs either in Asia or Europe, which indicated that the porcine* KIT* gene was undergoing a purifying selection. Coat colour has undergone both natural selection and human selection during evolution. However, the effects of different selections are generally very slight at the level of nucleotide sequence variation. However, it is crucial to point that the polymorphisms analysis was limited by the small sample size in our study.

### 3.3. Phylogenetic Clustering of Wild Boars and Domestic Breeds

**Figure 2:**
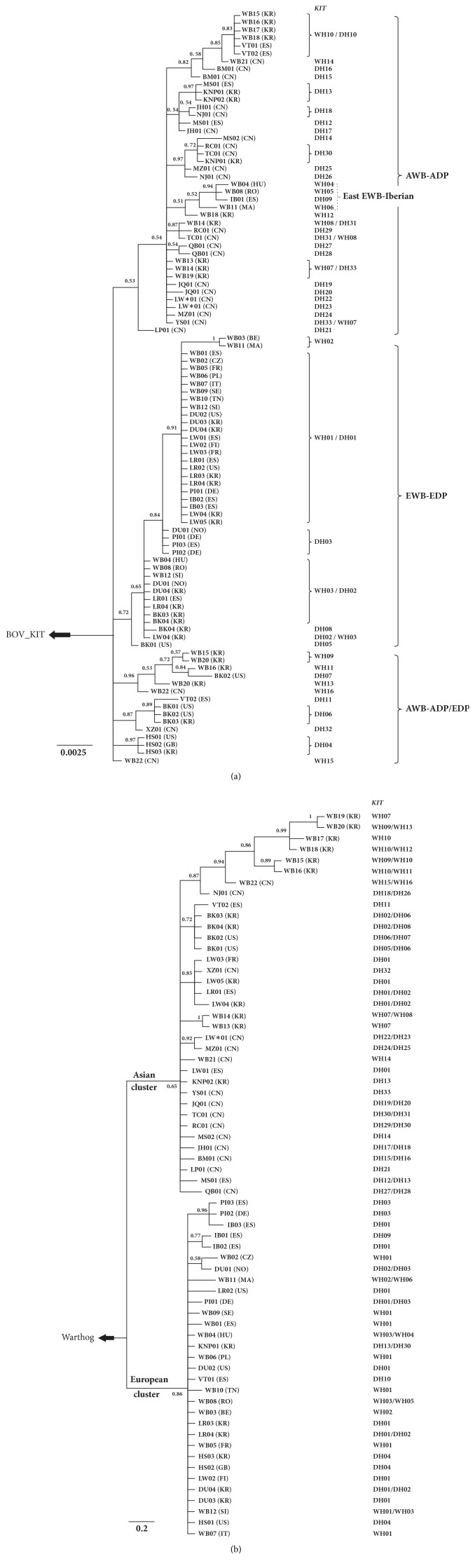
A consensus tree reconstructed for the investigated pigs using coding sequence of the* KIT* gene (a) and D-loop sequence (b).

A consensus tree was generated based on the estimated haplotypes of the investigated samples, as well as the one out group sequence (*Bos taurus*, NM_001166484). Two major and two minor clades were identified in the consensus tree using the mutations of the porcine* KIT* gene (Figure 2(a)). Majority of the Asian haplotypes clustered to clade 1 (AWB-ADP), except clade 4 (AWB-ADP/EDP) which consisted of five Asian wild boar haplotypes and four domestic haplotypes (BK: DH06-07, VT: DH11 and XZ: DH32). Almost all of the European haplotypes found in wild boar and domestic pigs were grouped together forming clade 3 (EWB-EDP). In addition, a small proportion of European haplotypes (WH04-06 and DH09) were identified as clade 2 (East EWB-Iberian), whereas the clade was clustered with clade 1. Subsequently, a neighbour-joining (NJ) tree was constructed using MEGA under a model of Kimura 2-parameters (Supplementary Figure 1), which gave similar topologies, with minor differences in terms of the different phylogenetic approaches. The values of bootstraps were quite low, which could be caused by low polymorphism among the whole coding region of the* KIT* gene (only 27 SNPs were detected among 2,919 base pairs).

**Figure 3:**
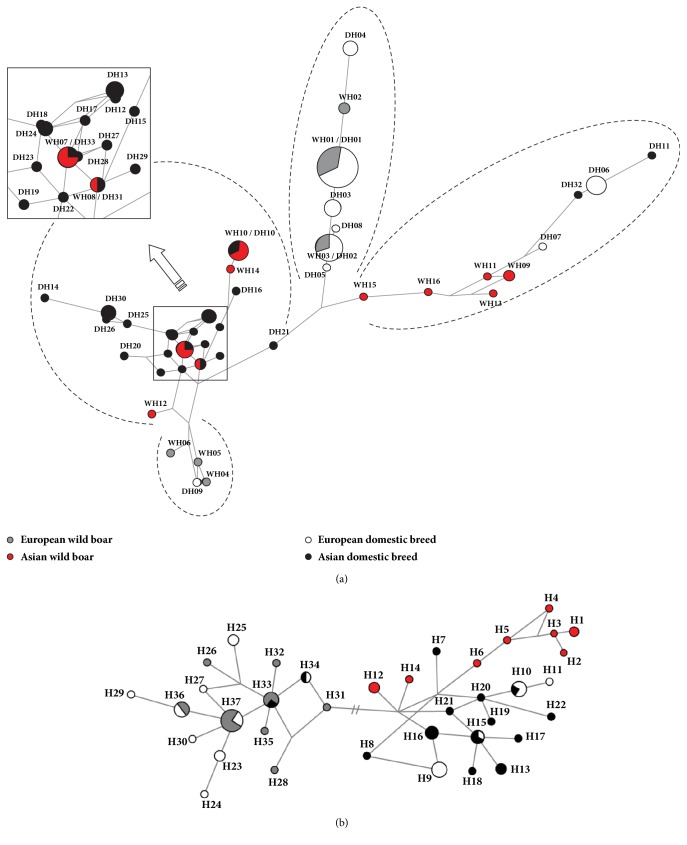
A parsimony median-joining network for the investigated pigs based on the* KIT* gene (a) and D-loop sequences (b).

A parsimony median-joining network was drawn using the haplotypes (Figure 3(a)). The AWB-ADP subgroup contains almost all Chinese domestic pigs except XZ and two KNPs. There are a total of 13 different Chinese domestic pigs in this study, and up to 22 haplotypes were inferred from Chinese pigs, which indicated a high genetic diversity in Chinese domestic pigs. There were 7 haplotypes presented in the EWB-EDP branch, which contained all the Landrace, Large White, Hampshire, Pietrain, and Duroc and 12 European wild boars. A unique group was AWB-ADPEDP, which are several “European-like” haplotypes from Chinese and Korean wild boars that contributed to 4 domestic haplotypes, which were mainly found in BK, XZ, and VT. Furthermore, three rare East European boars' haplotypes and an Iberian-specific haplotype were found in the last groups, which were in close proximity to the AWB-ADP group. Four groups were clearly identified and supported the result of phylogenetic tree.

### 3.4. mtDNA Is Consistent with the Divergent Evolution of the KIT Gene

The phylogenetic analysis of the D-Loop region of wild boars displayed two distinct clades: one Asian and one European (Figure 2(b)). The Asian clade included Korean wild boars, Chinese wild and domestic pigs, and three European domestic pigs (Landrace, Large White, and Berkshire). The most likely explanation for the three European breeds containing the Asian haplotype is that there would have been some introgressions in Asian pigs during their breeding histories. It was confirmed in written records and contemporary art that there were introgressions of Asian pigs during the 18th and early 19th centuries [29]. Furthermore, Chinese haplotypes have affected the haplotype composition of European pigs, such as Landrace (12%) and Large White (76%) [31]. The European clade was composed of European wild and domestic pigs, one potbelly pig from Vietnam (VT01) and one Korean native pig (KNP01). VT01 was clustered with the European clade, which could be explained by the raising of Vietnamese domestic pigs in Spain, which would thus cause some introgressions with the European samples. Unexpectedly, KNP01 were grouped with three European wild boars, which shared the same mtDNA haplotype (H33). The reason might be for European maternal inheritance in KNP during the early breeding history [32]. The mtDNA distinct clades supported the independent domestication events of pigs and were consistent with the independent evolution of the* KIT* gene in Asia and Europe, even though there were several introgressions in European and Asian pigs. As elucidated in phylogenetic trees and network analyses of the* KIT* gene, Landrace, Large White, and Berkshire have a clear Asian genetic background (Figures 2 and 3).

In this study, the Asian wild boars carried two major types of haplotypes (one Asian and one European), while the European wild boars contained one major haplotype and a small portion of other haplotypes (East European type). Since these patterns were revealed within a few wild boars, they may not lead to a scientific conclusion. We confirmed these patterns by a large sample set containing 37 European wild boars and 42 Korean wild boars (Figure 3(b)). The haplotypes of Asian wild boars were classified into two separated parts: the Asian group (WH07, WH08, WH10, WH12, and WH14), which forms the gene pool for Chinese and Korean domestic pigs, and the European group (WH09, WH11, WH13, WH15, and WH16), which contributed to the formation of Large White, Landrace, Pietrain, Hampshire, and Duroc breeds. All the European commercial breeds clustered within the European wild boar group, even though many of them accumulated more mutations. However, one Chinese domestic pig (XZ) clustered with the European pigs, which may have been caused by either of the two major reasons: one possible reason is crossbreeding imported European pigs with domestic Chinese pigs; the other possible cause is random mutations. The phylogenetic tree and network analyses revealed 4 subgroups in the investigated pigs, which was a clear divergence between domestic pigs in Asia and Europe. Both groups were domesticated independently in corresponding locations, even though there were some introgressions in recent history. The result obtained by mtDNA analysis is similar. As the mtDNA data revealed, modern domestic pigs had multiple originations and were subsequently domesticated [29].

### 3.5. The Relationship between KIT Genotypes and Coat Colour

Up to now, 5 alleles have been identified in the Dominant White locus (*i*,* I*,* I*^*P*^,* I*^*Be*^, and* I*^*Rn*^) [7, 11, 33, 34]. Furthermore, a large number of mutations were accumulated during the evolutional process either in coding regions or noncoding regions (many SNPs were found in intron4 and intron5, in the roan paper) [35]. None of these SNPs were identified as the causative mutation for the corresponding phenotype except the splice mutation in intron17 (G to A substitution), which contributed to the Dominant White phenotype with a normal copy of the* KIT* gene [5]. A* KIT* gene duplication mutation associates with a partially dominant phenotype (patch), and one exon skipping leads to the roan phenotype [36], while it is assumed that the basis of the belt phenotype was caused by an unknown regulatory mutation [7].

## 4. Conclusions

In conclusion, we found that the phylogenetic analysis of the Dominant White locus (*KIT*) indicated four geographic clusters, which supported the multiorigins of modern pigs. Furthermore, both Asian and European domestic pigs evolved under purifying selection during their evolutionary histories.

## Figures and Tables

**Table 1:** Primer sets used in this study.

Primer Name	Sequence (5'-3')	Product size (bp)	TM (°C)
KIT_E1F	GGAACGTGGAAAGGAGCT	205	66
KIT_E1R	CAGCCACCTCTTCTGACAC		
KIT_E2F	ACCCTTGCACCATAAATAGC	498	59
KIT_E2R	GACTCAGCCATCTATATGTC		
KIT_E3F	TCAGATTCGTTAACCACTGC	447	64
KIT_E3R	TGGCATATCCGCTGATCTA		
KIT_E4F	CGAAAGGCCATACAGTGAG	362	50
KIT_E4R	TGTATGAAAGCAACTATACC		
KIT_E5F	TCCCCCCGGGACTGCAGAGATTTGGGAATTATG	548	59
KIT_E5R	CGCGGATCCTCAACCTACTACTTCTAAGGTGG		
KIT_E6F	GAAGACATAAGATGGTGATA	385	53
KIT_E6R	CTAATATCCATGAGGACGCAGG		
KIT_E7F	CCAAAGCAGGCGTGTATTT	344	55
KIT_E7R	GGTCAAAATTATCATGAAGCAG		
KIT_E8F	GTCATCTTCAGCCTCAAGAAAG	313	54
KIT_E8R	TCTGAAAACATATTTGAAATTC		
KIT_E9F	GGCAAATAATTTTTCTTCTAG	293	54
KIT_E9R	CAGGCAGAGCCTAAACATCC		
KIT_E10-11F	CTTGACTCCTGCCATGATG	536	64
KIT_E10-11R	CTAGAACAAAGGAAGCTACCG		
KIT_E12-13F	GTCCCTGATTCCTTTATTGG	486	60
KIT_E12-13R	GCTTATCATCAAGGATGGTC		
KIT_E14F	GCATGTGAATGGCCGTGATC	322	50
KIT_E14R	ATCAGCCTTGATTGCAAACC		
KIT_E15-16F	CAAAGTCAGCCTCTTATGTGA	910	52
KIT_E15-16R	CTACGGCTCTAAAATGCTCC		
KIT_E17F	TGGCACCATATAACATAGGC	325	60
KIT_E17R	GGTGTGCATTATGAAACTCAC		
KIT_E18-19F	GCAGCAGGAGCAGTATCTAC	482	63
KIT_E18-19R	AACCTTCAACATCTGGGTTC		
KIT_E20F	GGTGTACACCCAAAGACAG	217	63
KIT_E20R	CTGTTCAAGGCGTTCCAAGC		
KIT_E21F	ACTTGCGATTCTGGACCTGC	271	63
KIT_E21R	GAATGGCAGTAGGTCGGTGC		

**Table 2:** Diversity and neutrality indices of *KIT* gene in the tested pigs.

Subgroups		No. of		No. of haplotypes	*π* ^#^	*θ* ^#^	Tajima's	Fu and Li's	
		ns	s				D	D^*∗*^	F^*∗*^
G1	AWB-ADP	3	14	24	1.68	1.56	0.28	-0.08	0.03
G2	EWB-EDP	3	8	7	1.63	1.54	0.16	0.16	0.17
G3	AWB-ADPEDP	3	12	9	2.21	1.89	0.81	0.79	0.89
G4	East EWB-Iberian	1	4	4	0.91	0.94	-0.21	-0.21	-0.20

Subgroups were identified by network analysis. ns: nsSNP (nonsynonymous single nucleotide polymorphism), s: sSNP (synonymous single nucleotide polymorphism), *π*: nucleotide diversity, and *θ*: Watterson's theta estimator; ^#^ per kilobase between sequences, within subgroup, wild boars' haplotypes found in domestic pigs and domestic pigs' haplotypes found in wild boars were excluded from neutrality analysis.

## Data Availability

The data used to support the findings of this study are available from the corresponding author upon request.

## Supplementary Materials

Supplementary Materials Supplementary 1.* Supplementary Figure 1*: a NJ tree constructed for the investigated pigs using 2,919 bp of* KIT* gene coding sequence. Supplementary 2.* Supplementary Table 1*: the information about the investigated pigs in this study.
